# Placental syndromes and long-term risk of hypertension

**DOI:** 10.1038/s41371-023-00802-4

**Published:** 2023-01-26

**Authors:** Abigail Fraser, Janet M. Catov

**Affiliations:** 1 Population Health Sciences, Bristol Medical School and the MRC Integrative Epidemiology Unit, University of Bristol, Bristol, UK; 2 Department of Obstetrics, Gynaecology and Reproductive Sciences and Epidemiology, University of Pittsburgh, Pittsburgh, PA USA

**Keywords:** Diseases, Pre-eclampsia

## Abstract

Higher blood pressure prior to pregnancy is associated with increased risk of placental abruption, hypertension and preeclampsia, preterm delivery and fetal growth restriction. These conditions are jointly termed placental syndromes as they are characterised by impaired placentation and early placental vascularization. Placental syndromes are associated with an increased maternal risk of progression to hypertension and cardiovascular disease (CVD) in later life. Women affected by both a clinical placental syndrome and with evidence of placental maternal vascular malperfusion (MVM) have a particularly high risk of hypertension and CVD. Yet whether placental impairment and clinical syndromes are causes or consequences of higher blood pressure in women remains unclear. In this review, we address the relationship between blood pressure and maternal health in pregnancy. We conclude that there is a pressing need for studies with a range of detailed measures of cardiac and vascular structure and function taken before, during and after pregnancy to solve the ‘chicken and egg’ puzzle of women’s blood pressure and pregnancy health, and to inform effective precision medicine prevention and treatment of both placental syndromes and chronic hypertension in women.

## Introduction

Maternal blood pressure decreases very early in the first trimester of pregnancy due to decreased systemic vascular resistance, reaching a nadir at mid-pregnancy after which blood pressure rises and returns to pre-pregnancy levels by term [1–3]. Women with hypertension prior to pregnancy are at increased risk of developing a placental syndrome. Placental syndromes include placental abruption, early and late-onset as well as super-imposed preeclampsia, fetal growth restriction, and preterm delivery, suggesting that pre-pregnancy blood pressure may contribute to all of these syndromes [4, 5].

The placenta provides essential nutrient and oxygen exchange to the growing fetus. Impaired placentation and early placental vascularization give rise to clinical placental syndromes; and each of these, in turn, is linked to a higher risk of hypertension after delivery [6–9]. In this review, we address the evidence as well as knowledge gaps about the links between pregnancy, placental syndromes, and maternal blood pressure, and why placental syndromes may be sex-specific markers of adverse life course blood pressure patterns.

## The effect of pregnancy on blood pressure

Several studies have shown that maternal blood pressure falls following pregnancy [10–13]. Using repeated blood pressure measures from pre-conception up to 40 years post-conception, we have shown that maternal systolic blood pressure (SBP) was −3.32 mmHg (95%CI:−3.93, −2.71) lower and diastolic blood pressure (DBP) −1.98 mmHg (95%CI:−2.43, −1.53) lower after a first pregnancy, with subsequent pregnancies having more modest effects. SBP and DBP changes from pre- to post- second pregnancy were −0.68 (95%CI −1.30, −0.06) and −0.31 (95%CI: −0.75, 0.14), respectively. For the third pregnancy, the results were: −0.24 (95%CI: −1.00, 0.52) and −0.59 (95%CI: −1.13, −0.06). We also found that it takes approximately a decade for parous women to ‘return’ to their pre-pregnancy blood pressure and that the difference between parous and non-parous women lasts beyond the menopause [10].

Similarly, a recent study used firstborn sex as an instrumental variable (or proxy) for number of children, exploiting the preference for sons in India. This preference for male offspring means that on average, women with a first-born daughter have a greater number of children than women with a first-born son. Authors found that parous women (but not men) with a first-born daughter had a -1mmHg (95%CI −1.26, −0.74) lower SBP and a −0.35 mmHg (95%CI: −0.52, −0.17) lower DBP than those with a first-born son [14]. This suggests that a greater number of pregnancies results in lower maternal – but not paternal – blood pressure. Whilst sex-specific effects of child-rearing on blood pressure cannot be completely ruled out, it is more likely that the inverse effect of family size on maternal blood pressure reflects child-bearing, i.e., pregnancy, effects.

## Clinical placental syndromes and blood pressure

Women with placental syndromes have approximately a two-fold risk of CVD and CVD-related mortality in later life [15–17] compared to women without a history of pregnancy complicated by a placental syndrome, and progression to chronic hypertension has been found to explain a large part of this increased risk. Indeed, European and U.S. preventive guidelines now recognize preeclampsia as a female-specific CVD risk factor [18–20]. A recent report using data from the US Nurses’ Health Study II, found that chronic hypertension explained 81% of the increased risk of CVD in women with a first pregnancy affected by gestational hypertension and 48% of the increased CVD risk associated with preeclampsia [15]. These findings are in line with previous reports using data from Norway [21] and the UK [22, 23].

There are three possible explanations for the increased risk of CVD associated with placental syndromes that is largely mediated by chronic hypertension as well as the drop in blood pressure seen post-pregnancy, and they are not mutually exclusive explanations. The first is that women with placental syndromes do not experience a drop in blood pressure after pregnancy like women without a placental syndrome either due to pre-pregnancy factors or as a consequence of the placental syndrome. The second is that women with placental syndromes do experience a comparable drop in blood pressure, but they have higher pre-pregnancy blood pressure. This would suggest that pregnancy is a natural ‘screening test’ that identifies women that have a more adverse cardiovascular health profile prior to pregnancy [24]. Finally, women with placental syndromes may experience a sharper rise in blood pressure in the post-partum years, which would suggest that placental syndromes causally affect blood pressure, through end-organ damage to vasculature or the kidneys, for example.

**Fig. 1:**
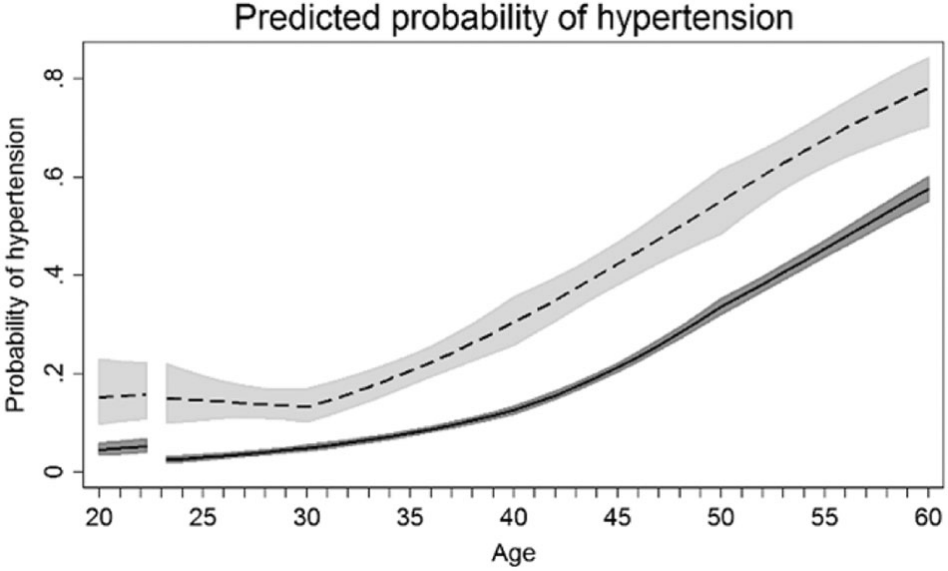
Population average predicted probabilities of hypertension defined as a current antihypertensive medication and/or blood pressure ≥140 mmHg systolic or ≥90 mmHg diastolic by age in women with normotensive and preeclamptic first pregnancies. Estimates are adjusted for age at measurement, HUNT survey, highest obtained education level, age at first birth, and ever daily smoking. Covariates are fixed at their means with gaps in the graphs corresponding to the woman’s first pregnancy, birth at age 23, and a three months postpartum period.

Evidence from a study using linked data from the population-based Norwegian HUNT study and the Medical Birth Registry of Norway shows a comparable drop in SBP following pregnancy in both women with and without hypertension in pregnancy. However, no equivalent drop in DBP in women with hypertension in pregnancy was observed. We also found that women who went on to develop hypertension in pregnancy had a 5 mmHg (95%CI: 3.2, 7.2) higher predicted SBP and a 3.5 mmHg (95%CI: 2.0, 5.0) higher predicted DBP at age 20, prior to pregnancy, compared with women who had a normotensive pregnancy [25]. Similarly, in a smaller preconception cohort study, women who developed preeclampsia or fetal growth restriction (*n* = 15) had lower cardiac output and cardiac index, and higher total peripheral resistance before pregnancy compared to women who did not have a placental syndrome (*n* = 205) [26]. Finally, in HUNT, the age-related increase in blood pressure was similar in women with and without hypertension in pregnancy so that by age 60 years, SBP was 9.0 mmHg (95%CI 6.2–11.8) higher and DBP was 2.8 mmHg (95% CI, 1.0–4.6) higher in women with preeclampsia. These findings are in line with the hypothesis that pregnancy is cardiometabolic stress test that identifies women with a greater propensity for CVD. Figure 1 (first published here [25]) shows the prevalence of hypertension in women with preeclampsia (the curve for women with gestational hypertension is virtually identical but not plotted for clarity) compared with women with a normotensive first pregnancy. By age 60 years, 78% and 79% of women with a first preeclamptic and hypertensive pregnancy respectively had hypertension, compared with 58% of women with a normotensive pregnancy.

Similarly, women with a first-born baby who was small-for-gestational-age (SGA) also had higher mean blood pressure across adulthood (from before to after pregnancy) than women with a first-born baby who was appropriate-for-gestational-age (AGA), despite having lower adiposity levels [27]. Interestingly, when we compared life-course trajectories of blood pressure and other cardiovascular risk factors in women with a first normotensive preterm pregnancy vs. term, there was no strong evidence that trajectories differed. This may be because normotensive pre-term delivery is a heterogeneous phenotype. Given that the placenta is a crucial organ of human pregnancy and its complications, it may reveal additional clues regarding the links between preterm delivery (and other placental syndromes) and maternal risk of hypertension and CVD, as discussed in the next section.

## Placental pathology, clinical placental syndromes, and blood pressure

A common finding in preeclampsia is the failure of the maternal decidua to adapt to adequately perfuse the placenta to support fetal growth and development [7]. This decidual vasculopathy includes insufficient remodelling of the uterine spiral arteries into highly dilated conduits feeding the placenta and related spiral artery pathologies. Decidual vasculopathy and concomitant hypoxia/reperfusion lesions in the placenta are collectively termed maternal vascular malperfusion (MVM) [7]. MVM is most classically and consistently detected in early-onset preeclampsia, early-onset fetal growth restriction, and in preeclamptic pregnancies delivered preterm or with accompanying fetal growth restriction [7, 28], suggesting that sub-types of clinical placental syndromes (e.g., early versus late onset preeclampsia) may be affected by different forms of placental impairment. MVM lesions are also present in up to a third of spontaneous preterm births [6], and retroplacental haemorrhage, the placental evidence of abruption, is also a diagnostic feature of MVM [29].

The causes of MVM are not known and are likely to be numerous; immunological and inflammatory components have been proposed, particularly in relation to acute atherosis [30, 31]. Another possible cause is pre-existing maternal predisposition to microvascular dysfunction, which is unmasked by the cardiometabolic stress test of pregnancy and apparent in the placenta. Regardless of its causes, MVM can result in both a poorly perfused placenta and circulating endothelial microparticles which themselves induce or exacerbate systemic vessel injury, perhaps via oxidative stress damage [32–34]. Thus, this initial damage may extend both spatially outside the placenta, and temporally outside of the pregnancy event. For example, MVM is a risk factor for preeclampsia in subsequent pregnancies, even in women without prior preeclampsia [35]. Abnormalities of the maternal microvasculature in other beds have also been described in women with preeclampsia with or without MVM, including capillary rarefaction in the fingernail [36] and skin [37–39], reduced venular diameters in conjunctiva [36], increased microvascular reactivity in skin blood flow, and abnormal endothelial glycocalyx of sublingual micro-vessels [35, 39–42]. There is also evidence that there are changes in the microvascular structure of the retina assessed via optical coherence tomography angiography (OCTA) during pregnancies complicated by preeclampsia compared to normal pregnancy and the non-pregnant state [43].

We have reported that women with MVM in the placenta have higher DBP (2.56 mmHg; 95%CI: 0.39, 4.74) but not SBP ten years after delivery compared to women with no evidence of vascular pathology in the placenta [44]. These findings were independent of pregnancy outcomes and maternal BMI. Of note, both SBP and DBP were higher 10 years post-delivery as the severity of the placental MVM worsened. In addition, MVM-affected women compared to those with a healthy placental vasculature had evidence of microvascular rarefaction and a more atherogenic lipid profile [44]. It also appears that only the subset of women with idiopathic preterm birth and MVM lesions have an excess risk for higher blood pressure and atherogenic lipids after delivery, both of which contribute to subclinical atherosclerosis after delivery [44, 45].

Consistent with these data, there is evidence that seven months after delivery, women with preeclampsia with accompanying decidual vasculopathy (the most severe, chronic, and likely underlying precursor lesion to other MVM features) had higher DBP, lower left ventricular stroke volume, and higher total peripheral vascular resistance compared with women with preeclampsia but no decidual vasculopathy [46]. A large, prospective, community-based U.S. cohort has also reported that mural hyperplasia, a feature of decidual vasculopathy characterized by thickening of the smooth muscle wall of the spiral arteries, is associated with risk of hypertension a decade after delivery when accompanied by modest BP elevations in pregnancy [47].

While even modest pre-pregnancy blood pressure elevations are associated with excess risk of placental syndromes during pregnancy, as shown above, there are sparse data directly examining the association of pre-pregnancy blood pressure and MVM occurrence. We have reported that the expected drop in DBP from pre-conception to early pregnancy (10 weeks, on average) was blunted in women who delivered placentas with evidence of MVM (−1.35 vs. −5.66 mmHg in women with no MVM). These data further support the possibility that subtle pre-existing vascular dysfunction may contribute to failed placental vascularization detected in placenta syndromes that is a marker for excess hypertension risk later in life.

## Preventative treatment following a placental syndrome

Studies assessing prevention and treatment options for placental syndromes during pregnancy understandably focus on short terms maternal and fetal outcomes and have recently been comprehensively reviewed by Magee et al. [48], with post-partum interventions and management strategies to mitigate CVD in women who experienced a placental syndrome also recently reviewed by Jowell et al. [49].

The SNAP-HT trial evaluated the feasibility of self-management of postpartum hypertension with automated medication reduction via telemonitoring in women requiring antihypertensive medication in the puerperium. Investigators reported improved blood pressure control and that DBP remained lower at six months post-partum in the intervention group compared to the control group (−5.4 mmHg; 95%CI: −8.1, −0.8). The difference in SBP between the two arms was more modest (−1.0 mmHg; −6.3, 4.4) [50, 51]. Results of the subsequent main trial (POP-HT) that is powered to detect differences in 24-hour average ambulatory diastolic blood pressure at 6–9 months post-partum, with secondary outcomes including multimodal cardiovascular assessments (CMR and echocardiography), parameters derived from multiorgan MRI including brain and kidneys, and peripheral macrovascular and microvascular measures are expected to be published in the near future [52].

## Summary

Hypertension and higher blood pressure prior to pregnancy are associated with increased risk of clinical placental syndromes and MVM, and those in turn, are associated with an increased risk of progression to hypertension and CVD in later life. Women with both clinical placental syndrome and evidence of placental MVM have a particularly high risk of hypertension and CVD. Both SBP and DBP are higher prior to pregnancy in women who go on to develop a placental syndrome and MVM, supporting the hypothesis that placental lesions and clinical syndromes are indicative of a pre-existing propensity for hypertension and CVD. Limited evidence suggests that DBP (but not SBP) fails to normalise following a hypertensive pregnancy, that the early pregnancy drop in DBP (but not SBP) is blunted in women with MVM, and that DBP is more amenable than SBP to post-partum treatment. DBP—more so than SBP—reflects microvascular dysfunction in young women, it is therefore possible that the latter plays a role in the pathogenesis of placental syndromes. It is also possible that placental syndromes further damage women’s microvasculature, directly contributing to the increased risk of hypertension post-partum. Studies with detailed measures of cardiac and vascular structure and function, from before to after pregnancy are needed to solve the ‘chicken and egg’ puzzle of women’s blood pressure and pregnancy health. Postpartum Intervention trials among women with placental syndromes can also help disentangle this question, and consideration of DBP as a primary endpoint is warranted.
