# Correction: Placental syndromes and long-term risk of hypertension

**DOI:** 10.1038/s41371-023-00860-8

**Published:** 2023-09-07

**Authors:** Abigail Fraser, Janet M. Catov

**Affiliations:** 1 Population Health Sciences, Bristol Medical School and the MRC Integrative Epidemiology Unit, University of Bristol, Bristol, UK; 2 Department of Obstetrics, Gynaecology and Reproductive Sciences and Epidemiology, University of Pittsburgh, Pittsburgh, PA USA

**Keywords:** Diseases, Pre-eclampsia

Correction to: *Journal of Human Hypertension* 10.1038/s41371-023-00802-4, published online 26 January 2023

In this article the ‘Funding’ statement was missing and should have been ‘This work was supported by the Medical Research Council [grant number MR/M009351/1].’

The original article has been corrected.

